# Study on the Growth and the Photosynthetic Characteristics of Low Energy C^+^ Ion Implantation on Peanut

**DOI:** 10.1371/journal.pone.0068769

**Published:** 2013-07-05

**Authors:** Yuguo Han, Lei Xu, Peiling Yang, Shumei Ren

**Affiliations:** 1 Key Laboratory of Soil and Water Conservation and Desertification Combating, Ministry of Education, School of Soil and Water Conservation, Beijing Forestry University, Beijing, China; 2 China Irrigation and Drainage Development Center, Beijing, People’s Republic of China; 3 College of Water Resources and Civil Engineering, China Agricultural University, Beijing, China; Lawrence Berkeley National Laboratory, United States of America

## Abstract

Employing the Nonghua 5 peanut as experimental material, the effects of low energy C^+^ ion implantation on caulis height, root length, dry weight, photosynthetic characteristics and leaf water use efficiency (WUE) of Peanut Ml Generation were studied. Four fluences were observed in the experiment. The results showed that ion implantation harmed the peanut seeds because caulis height, root length and dry weight all were lower in the treatments than in CK, and the harm was aggravated with the increase of ion fluence. Both P_n_ and T_r_ show a saddle-shape curve due to midday depression of photosynthesis. Low fluence of low energy C^+^ ion implantation could increase the diurnal average P_n_ of peanut. The diurnal variation of T_r_ did not change as significantly as Pn. The light saturation point (LSP) was restrained by the ions. After low energy C^+^ ion implantation, WUE was enhanced. When the fluence increased to a certain level, the WUE began to decrease.

## Introduction

Low-energy ions exist widely in the natural world and are generated in the laboratory through acceleration, mass selection, transfer of energetic ions to a reaction chamber, and interaction with ion-target [1], [2], [3], [4]. Besides modifying semiconductor surfaces, low-energy ions modify the genes of plants and microorganisms and have represented a novel and effective method for genetic modification in recent decades. Although there is no consensus on the lethality rate of different low-energy ions (C^+^, N^+^, Ar^+^ and H^+^) in breeding plants and microbes, it is clear that C^+^ induces a higher mutation rate and has been applied to breeding of rice, tomato, cotton, sweet potato, maize, wheat, buckwheat, rose, and carnations [5], [6], [7], [8], [9], [10], [11], [12], [13], [14], [15]. In the mid 1980 s, the biological effects in organisms induced by keV ions were recognized and demonstrated experimentally. The relationship between low-energy ions and the organism occurs at the physical and chemical level as well as biologically. The method has four advantages: (1) high variation rate; (2) wide genetic variation genealogy; (3) quick and stable variation; and (4) no radioactive contamination [16], [17], [18].

The peanut (Arachis hypogaca L.) plays an important role in economic development and agricultural production in China, and research on how to improve quality and stress resistance of peanut are of great importance for enhanced production. Although ion implantation, as a new technology, has been applied to breeding of many plant species, it has seldom been applied in peanuts [19], [20]. Currently, researchers in the area of ion implantation in plants focus on the change in the biological characteristics of the plant caused by the implantation and less on the physiological consequences e.g. drought resistance. The investigation on effect of the ion implantation technology on enhancing drought resistance is not only significant for water conservation in agriculture but also essential for determining whether or not the ion implantation technology can be applied to agricultural improvements. This paper studies the effects of ion implantation on biological and photosynthetic characteristics of peanut and discusses the drought resistance of peanut caused by ion implantation.

## Materials and Methods

No specific permits were required for the described field studies. The location was not privately-owned or protected in any way and the field studies did not involve endangered or protected species.

### Experimental Material

Nonghua 5 peanut was used as the experiment material and was supplied by China Agricultural University. Nonghua 5 peanut is rich in protein and has a thin skin, so it is suitable for low energy C^+^ ion implantation. The ion species was C^+^ with the low energy of 40 keV. The seed coats had been removed before ion implantation. The ion was implanted by an ion implanter into the embryo (radicel part) of peanut seed using the pulse implantation technique at the Institute of Low Energy Nuclear Physics in Beijing Normal University. Based on different fluences, there were four treatments: treatment I: 1×10^11^ C^+^·cm^−2^, treatment II: 1×10^12^ C^+^·cm^−2^, treatment III: 1×10^15^ C^+^·cm^−2^ and treatment IV: 1×10^16^ C^+^·cm^−2^, and CK: 0 C^+^·cm^−2^. Five hundred seeds were chosen for each treatment and all of them were plump, uniform in size and dried by air. During ion bombardment, the temperature was no more than 30°C, the pressure was kept around 10^−5^ Pa by a turbomolecular pump, and the beam current was below 10 µA·cm^−2^, so any rise in temperature could be ignored and the seeds would not be carbonized. Finally, seeds with an intact appearance were selected for planting.

### Planting Method

The peanut seeds were planted in the southeast area of Pangge Village in Daxing district of Beijing. The soil type was sandy loam. The average dry density was 1.38 g cm^−3^. In the soil, the total nitrogen was 0.058%, the average N, P, K contents were 0.005%, 0.002% and 0.013%, respectively, and the soil organic matter content was 0.95%. Thus, this land was suitable for the growing of peanut plants.

The seeds were planted in the middle of May in 2008 and this experiment was conducted in a completely randomized design. Each experiment section was 2.1 m×2.0 m in size, 3 times repeated. The seeding method was hill-drop. The hill spacing was 20 cm, and the row spacing was 35 cm. The results were compared with those in CK in which no low energy C^+^ ion was implanted.

The germination potential and the budding rate were calculated by statistical method on the 4th day and the 10th day after planting, respectively. The seedlings were taken out from the soil and cleaned on the 20th day. The dry weight of the root and shoot were measured by surveying and weighing.

### Measurement Method

At the 20th day, 20 seedlings were taken out from each of the treatments and cleaned. Their caulis height, root length, shoot dry weight and root dry weight were measured from each seedling and averaged.

Caulis height is the length of the node between the cotyledon and the uppermost unrolled leaf from each seedling. Root length is the sum of primary root length and lateral root length from each seedling as an average. Shoot dry weigh and root dry weight are respective weights of the above-ground and below-ground parts of each seedling.

Photosynthetic characteristics were collected with the LI-6400 (LICOR,Inc. Lincoln, NE, USA) Photosynthesis System in the flowering-pegging and pod setting stages over three sunny days. The measurement was conducted between 9am and 5pm with an interval time of 2 hours. The fourth pinnately compound leaf was chosen to measure the parameters, photosynthesis (P_n_), transpiration (T_r_), leaf temperature, air humidity, photosynthetically available radiation, etc., with each measurement repeated 3 times and their average value used as observation data.

WUE represented the water use efficiency of leaves, which was calculated using the following equation:
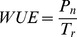



### Data Analysis

The data were analyzed with the software of SPSS12.0 [21].

## Results and Discussion

### Caulis Height and Root Length

**Figure 1:**
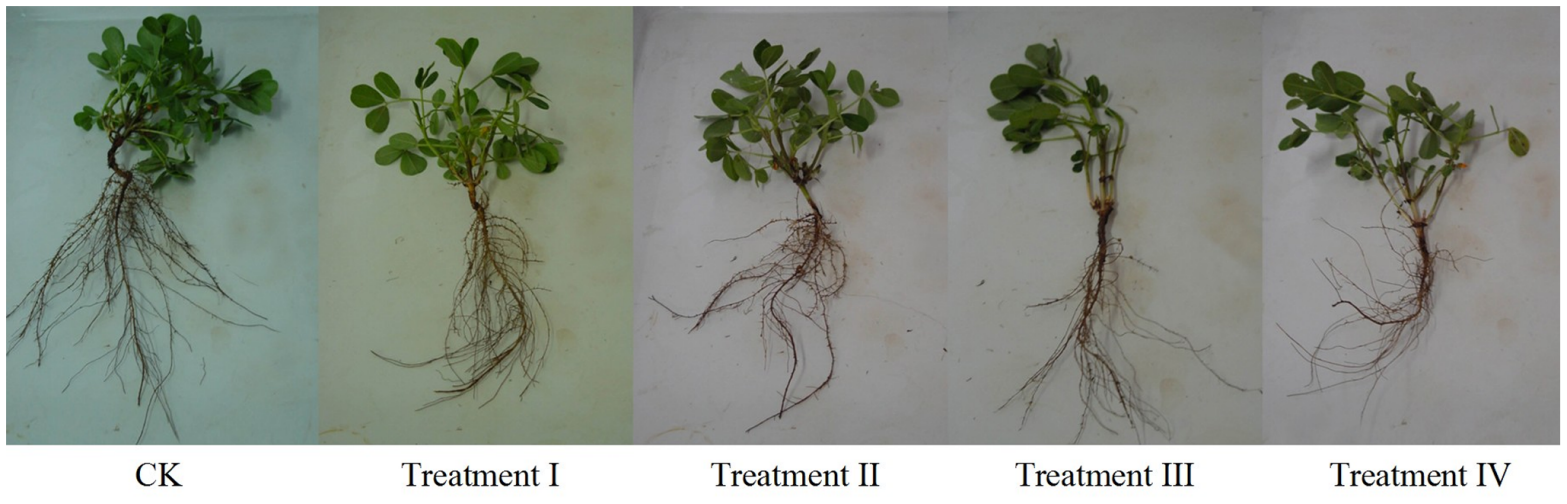
Images from different treatments in seedling stage.

In Table 1 and Fig. 1, both caulis height and root length declined after ion implantation. The lower the fluence of ions (e.g., in Treatment I), the slighter the biological harm to the seedling, and the less their root length decreased. With the increase of the fluence, the biological harm caused by low energy C+ ion implantation to the seedlings resulted in a rapid reduction in their root lengths. When the root length was reduced to a certain degree, its rate of reduction diminished with increasing fluence. Therefore, with the increase in fluence, the effect of ion implantation on the peanut M1 generation is more significant and the biological harm caused by ion implantation is also aggravated.

**Table 1:** Caulis height and root length of seedlings from different treatments.

	Treatment I	Treatment II	Treatment III	Treatment IV	CK
Caulis height (cm)	10.60	9.60	8.90	8.50	10.80
Root length (cm)	246.39	136.14	121.06	118.14	282.06

### Dry Weight of the Root and Shoot in Seedling Stage

**Figure 2:**
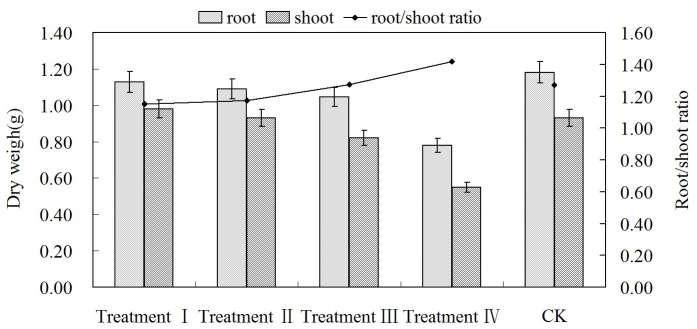
Effect of fluence on the dry weight and root-shoot ratio in seedling stage.

According to Fig. 2, low-energy ion implantation resulted in a change of biomass accumulation in peanut seedlings and inhibited the growth of their shoot and root. Therefore, both shoot dry weight and root dry weight were reduced to various degrees, and similar to caulis height, the degree of their reduction increased with the increase of the ion fluence.

In Fig. 2, root/shoot ratio increased as the fluence increased. It indicates that as fluence is lowered there is reduced harm to the peanut seed and less impact on root growth; thus the root can supply the shoot with adequate moisture and nutrients to maintain normal growth of the plants. With gradual increase of the ion fluence, the harm to the peanut seeds gradually increases because the dry weight accumulation is declining in both root and shoot. As the harm increased, the root grew more slowly. Therefore, water and nutrient supply to the shoot was reduced and finally growth of the shoot was constrained, leading to an objective increase in the root/shoot ratio.

The parameters of M1 generation seedlings were statistically analyzed with SPSS software (Table 2).

**Table 2:** F test of the variance analysis of M_1_ generation characteristics.

	Caulis height	Root length	Root dry weight	Shoot dry weight
F	12.996	192.465	32.355	45.105
Sig	0.001	0.000	0.000	0.000

“F” is a statistical verification value of F-distribution. “Sig” is a value of statistical significance, which indicates the probability of occurrence of the experimental result.

With different ion fluences, caulis height, root length, root dry weight and shoot dry weight all had a significant value (Sig) of less than 0.05. These results indicate that change of ion fluence has a significant effect on seedling growth of peanut M1 generation.

After implanted into peanut seeds, low-energy C-ions collide directly with DNA in the seed cells. After a series of complicated biological, physical and chemical changes, chromosomes within the plants were damaged, and cell mitosis becomes abnormal. As a result, caulis height and root length were changed.

Genetic mutation was processed from the inside to the outside, which resulted in and was indicated by a change of physiological characteristics. Xu, et al. also carried out a similar study on the biological effect of low energy C+ ion implantation on peanut seedlings of M1 generation. Their study showed that the germination potential and the budding rate reduced 10%–29.33% and 6%–24% respectively at the prime stage of the lifecycle [22]. In our experiment, the interrelationship between various physiological characteristics was investigated based on experimental observation and data analysis in combination with the previous research information, and caulis height, root length and dry weight were selected for assessment of the biological effect of low energy C+ ion implantation on peanut seedlings of M1 generation as plant height is an external manifestation of the plant’s growth ability, and growth state of the plant is directly dependent on the integrated development of all organs including root.

### photosynthetic Characteristics

**Figure 3:**
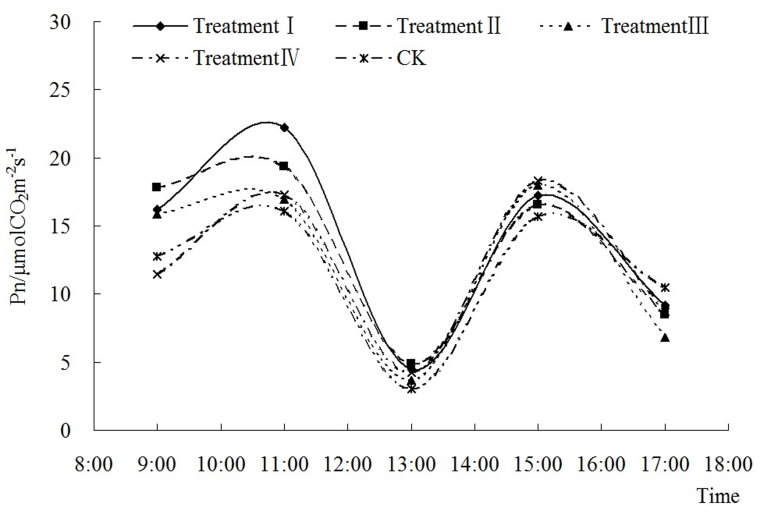
Diurnal variation of different fluences on photosynthetic rate in Nonghua 5 at flowering-pegging stage.

**Figure 4:**
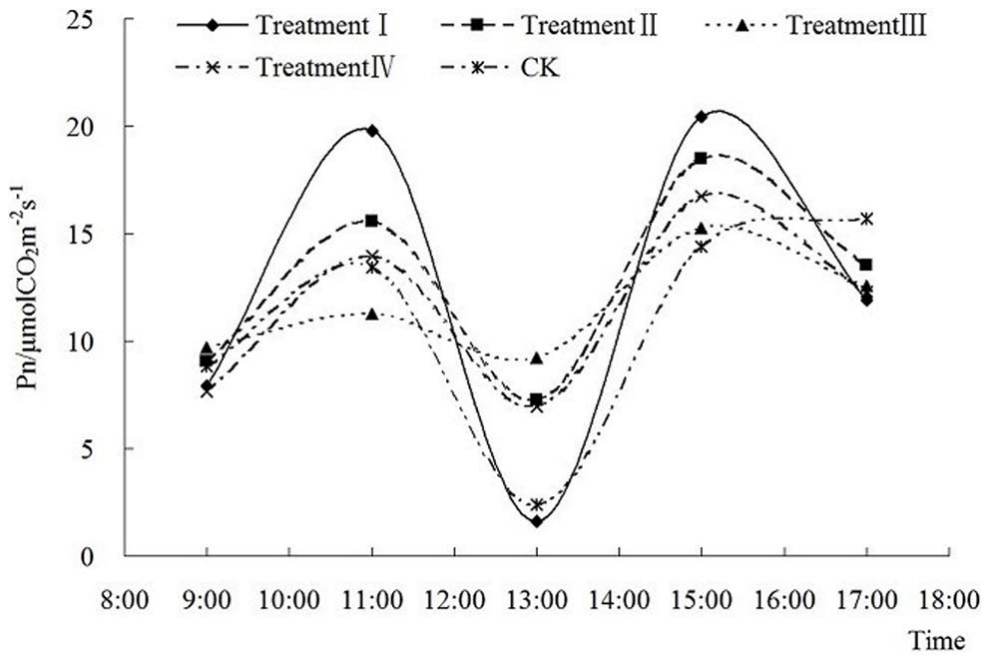
Diurnal variation of different fluences on photosynthetic rate in Nonghua 5 at pod setting stage.

**Figure 5:**
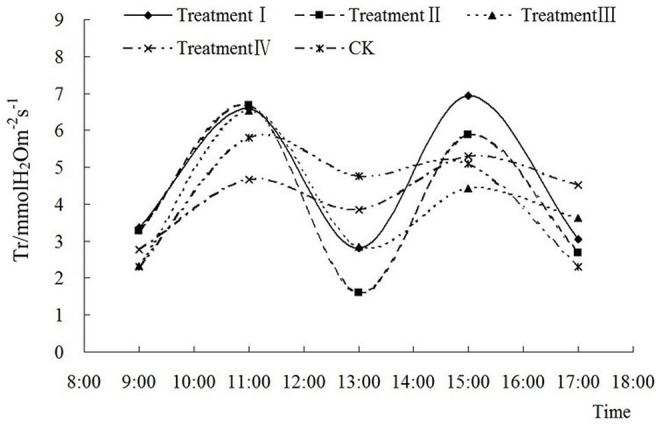
Diurnal variation of different fluences on transpiration rate in Nonghua 5 at flowering-pegging stage.

**Figure 6:**
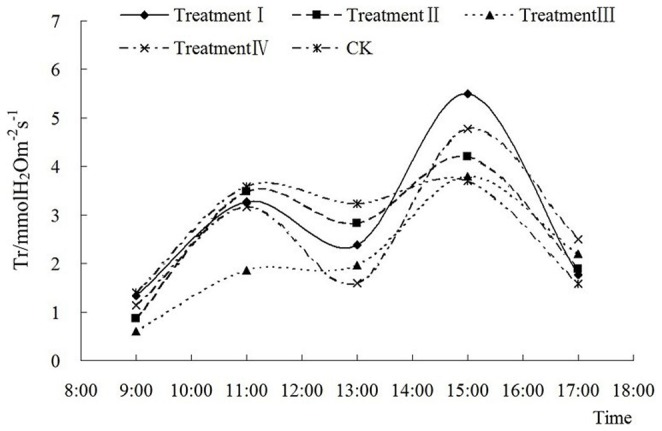
Diurnal variation of different fluences on transpiration rate in Nonghua 5 at pod setting stage.

Both P_n_ (Figs. 3 and 4) and T_r_ (Figs. 5 and 6) show a saddle-shape curve due to midday depression of photosynthesis. The midday depression of photosynthesis was principally caused by decline of leaf water content and stomatal conductance due to the fall of atmospheric humidity. The reduction of CO_2_ concentration also contributes to photosynthetic depression at midday. The midday depression of photosynthesis is a common phenomenon in C3 plants including peanut.

In Figs. 3 and 4, low energy C^+^ ion implantation did not change the diurnal variation trend of P_n_. In the flowering-pegging stage (Fig. 3), P_n_ increased significantly and then decreased with the increase of the ion fluence before 13∶00, but after 13∶00 the difference between the treatments decreased. In the pod setting stage (Fig. 4), the diurnal variation of P_n_ in CK and treatment I was significant. With the fluence increasing, the diurnal variation of P_n_ in treatments II, III and IV was lower and smoother. Compared with CK, the diurnal variations of P_n_ in four different treatments increased 19.38%, 5.50%, 5.51% and 3.36%, respectively in the flowering-pegging stage, and 12.60%, 16.71%, 6.03% and 5.21%, respectively in the pod setting stage. At 13∶00, P_n_ was 1.46 times, 1.58 times, 1.20 times and 1.39 times that of CK respectively in different treatments in the flowering-pegging stage, and in pod setting stage it was 0.68 times, 3.04 times, 3.86 times and 2.90 times that of CK respectively.

The experimental data indicated that after low energy C^+^ ion implantation, the diurnal average photosynthetic ability and the midday photosynthetic ability were both strengthened significantly (except in treatment I in the pod setting stage). This means that the low energy C^+^ ion implantation of peanut seeds affected the physiological rule, enhanced the photosynthetic ability, decreased the “midday depression” depth and raised the solar energy utilization efficiency. However, the changing trends vary with different times, fluences and bearing stages. To sum up, in different bearing stages and with different fluences, the diurnal average P_n_ was always higher in the treatments than in CK.

T_r_ had the same diurnal variation as a saddle shape curve in Figs. 5 and 6 as P_n_, which indicated that low energy C^+^ ion implantation also did not change the trend of diurnal variation of T_r_.

In the flowering-pegging stage (Fig. 5), the differences between peak and bottom values of T_r_ in treatments I, II, III and IV were 4.13 mmol H_2_Om^−2^s^−1^, 4.61 mmol H_2_Om^−2^s^−1^, 3.07 H_2_Om^−2^s^−1^ and 1.61 H_2_Om^−2^s^−1^, respectively, which were all higher than that in CK, 1.04 H_2_Om^−2^s^−1^. Therefore, the diurnal variation of T_r_ changed significantly after ion implantation in the flowering-pegging stage, but with the increase of fluence, this phenomenon became indistinguishable. In the pod setting stage (Fig. 6), the diurnal variation of T_r_ also showed a saddle shape curve, but had little difference to that observed in the flowering-pegging stage. Except in treatment II, there was variance between the peak and bottom values of T_r_. For instance, the difference between the peak and bottom values in treatment I was only 0.35 H_2_Om^−2^s^−1^ in the flowering-pegging stage but it reached 2.23 H_2_Om^−2^s^−1^ in the pod setting stage. Therefore, low energy C^+^ ion implantation enhanced the afternoon transpiration ability.

**Figure 7:**
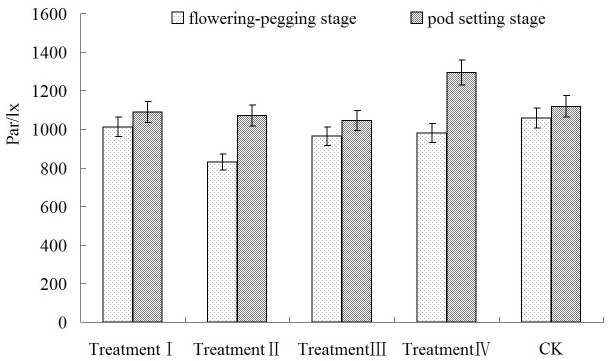
PAR at different growth stages on LSP.

In this experiment, the first peak value was caused by LSP (Light Saturation Point) in the diurnal variation of P_n_ in Figs. 3 and 4. Fig. 7 showed the values of PAR (light intensity) when each treatment reached LSP. Except treatment IV in pod setting stage, the value of P_n_ was always lower than that in CK when each bearing stage reached LSP. When each treatment reached LSP, the decrease in PAR ranged from 3.70% to 20.40% in the flowering-pegging stage, and from 3.81% to 7.36% (except in treatment IV) in the pod setting stage. In treatment IV, PAR increased by 14.36% compared with CK. Therefore, the difference in the treatments did not reach a significant level except in treatment II in the flowering-pegging stage. After low energy C^+^ ion implantation, the increase of LSP in Nonghua 5 peanut was restrained.

### Water Use Efficiency

WUE is an important parameter indicating drought resistance of plants. In both stages of flowering-pegging and pod setting, the differences of WUE in all treatments and CK were significant and the WUE of leaves rose with increasing fluence (Table 3). However, when the fluence increased to a certain level, the WUE would begin to decrease. Thus, there is a critical fluence effect between Treatment II and Treatment III where the difference in fluence was 1000 times. Therefore, it is feasible to use ion implantation technology to change WUE. The change in drought resistance caused by different fluences needs to be further studied. In addition, as ion implantation may affect more than one generation and the crop has self-recovery function, it is necessary to continue the study on M3 or further generations. It is significant to assess the persistence of the effect of ion implantation on crop drought resistance and to investigate the depth of the effect.

**Table 3:** F test of WUE in flowering pegging stage and pod setting stage.

Treatment	I	II	III	IV	CK	F	Sig
Flowering pegging stage %	3.05	3.67	3.71	2.56	2.87	8.89	0.031
Pod setting stage %	4.33	4.82	5.58	4.36	4.06	11.44	0.029

“F” is a statistical verification value of F-distribution. “Sig” is a value of statistical significance, which indicates the probability of occurrence of the experimental result.

With different ion fluences, WUE had a significant value less than 0.05. Therefore, change of ion fluence has a significant effect on seedling growth of peanut M1 generation.

### Conclusions

Fluence is a very important factor when ions are implanted into seeds as it directly affects seedling growth. In this research, ion implantation harmed peanut seeds. Caulis height, root length and dry weight were all lower in treatments of ion implantation compared to CK. With increase of ion fluence, the harm was also aggravated. Both P_n_ and T_r_ show a saddle-shape curve due to midday depression of photosynthesis. After low energy C^+^ ion implantation, the diurnal variation of P_n_ changed significantly. Low fluence of low energy C^+^ ion implantation could increase the diurnal average P_n_ of peanut, and the increasing degree declined with increasing of fluence. In this experiment, the diurnal average P_n_ was always higher in the treatments than in CK. The diurnal variation of T_r_ did not change as significantly as P_n_. The variance analysis suggests that the difference in diurnal average value of T_r_ was significant between the four treatments and CK. The LSP of peanut declined after low energy C^+^ ion implantation. Except treatment IV in the pod setting stage, the LSP increased 14.36% compared with CK, and the values of LSP all declined to some degree. The difference was between 3.70% and 20.40% in the flowering-pegging stage and between 3.81% and 7.36% in the pod setting stage. Treatment II showed a significant reduction in the flowering-pegging stage. After low energy C^+^ ion implantation, WUE was obviously enhanced, and the values of WUE in treatments I and II in bearing stages increased significantly compared with CK. When the fluence increased to a certain level, the WUE began to decrease.
