# Correction: Study on the Growth and the Photosynthetic Characteristics of Low Energy C^+^ Ion Implantation on Peanut

**DOI:** 10.1371/annotation/a47d116c-6475-45b4-8e40-167442da28b6

**Published:** 2013-07-09

**Authors:** Yuguo Han, Lei Xu, Peiling Yang, Shumei Ren

The following statement is missing from the Funding Information:

This work was funded and supported by Beijing Forestry University Young Scientist Fund (No. BLX2011009).

The following statement is missing from the Acknowledgments:

I would like to express my gratitude to Professor Tao Zhang for his help in ion implantation process from the College of Nuclear Science and Technology, Beijing Normal University. 

